# Estimating Y-Str Mutation Rates and Tmrca Through Deep-Rooting Italian Pedigrees

**DOI:** 10.1038/s41598-019-45398-3

**Published:** 2019-06-21

**Authors:** Alessio Boattini, Stefania Sarno, Alessandra M. Mazzarisi, Cinzia Viroli, Sara De Fanti, Carla Bini, Maarten H. D. Larmuseau, Susi Pelotti, Donata Luiselli

**Affiliations:** 1 Dipartimento di Scienze Biologiche, Geologiche e Ambientali (BiGeA), Università di Bologna, 40126 Bologna, Italy; 2 Dipartimento di Scienze Statistiche “Paolo Fortunati”, Università di Bologna, 40126 Bologna, Italy; 3 Dipartimento di Scienze Mediche e Chirurgiche, Università di Bologna, 40126 Bologna, Italy; 4 Laboratory of Forensic Genetics and Molecular Archaeology, Forensic Biomedical Sciences, KU Leuven, B-3000 Leuven, Belgium; 5 Laboratory of Socioecology and Social Evolution, Department of Biology, KU Leuven, B-3000 Leuven, Belgium; 6 Dipartimento di Beni Culturali, Università di Bologna, 48121 Ravenna, Italy

**Keywords:** Haplotypes, Population genetics, Genetic markers

## Abstract

In the population genomics era, the study of Y-chromosome variability is still of the greatest interest for several fields ranging from molecular anthropology to forensics and genetic genealogy. In particular, mutation rates of Y-chromosomal Short Tandem Repeats markers (Y-STRs) are key parameters for different interdisciplinary applications. Among them, testing the patrilineal relatedness between individuals and calculating their Time of Most Recent Common Ancestors (TMRCAs) are of the utmost importance. To provide new valuable estimates and to address these issues, we typed 47 Y-STRs (comprising Yfiler, PowerPlex23 and YfilerPlus loci, the recently defined Rapidly Mutating [RM] panel and 11 additional markers often used in genetic genealogical applications) in 135 individuals belonging to 66 deep-rooting paternal genealogies from Northern Italy. Our results confirmed that the genealogy approach is an effective way to obtain reliable Y-STR mutation rate estimates even with a limited number of samples. Moreover, they showed that the impact of multi-step mutations and backmutations is negligible within the temporal scale usually adopted by forensic and genetic genealogy analyses. We then detected a significant association between the number of mutations within genealogies and observed TMRCAs. Therefore, we compared observed and expected TMRCAs by implementing a Bayesian procedure originally designed by Walsh (2001) and showed that the method yields a good performance (up to 96.72%), especially when using the Infinite Alleles Model (IAM).

## Introduction

Y-chromosomal Short Tandem Repeats (Y-STRs) are important tools for forensic investigations, genetic genealogical applications, as well as for molecular anthropology and population genetics^1–5^. This is due to the unique, largely non-recombining properties of Y-chromosome, that is transmitted strictly along the paternal line in a virtually unaltered fashion, and the only factor causing variation between generations is the (rare) occurrence of mutations. In addition, it is characterized by smaller effective population size and more marked geographic differentiation with respect to autosomal markers, thus providing stronger phylogeographic signals and evidence of different patterns of genetic variation among human populations^6,7^. In most human populations Y-chromosome geographic specificity is also higher than that of its maternal counterpart, mitochondrial DNA. However, this is not a general rule, since there are marked differences in patterns of mitochondrial DNA and Y-chromosome variation between matrilocal and patrilocal populations^8^.

All these features, combined to the recent advances in sequencing technologies, guarantee to Y-chromosome studies an important role even in the genomics and post-genomics era. Potential applications range from reconstructing male-mediated migration events in the past, Y-chromosomal variation from ancient DNA, the impact on genetic variability of male-related social-cultural features (such as patrilineal systems of land inheritance, male-related social stratification, etc.), the extra-pair paternity behavior among human populations and of course forensics^9–14^.

The most important applications of Y-STRs in forensic investigation, such as paternity tests, individual identification and familial searching, rely on the possibility of tracing a relationship between two or more men and on the distinctiveness of individual haplotypes^3^. The same properties also apply to molecular anthropology, in which Y-STR variability can be used to estimate the relationships between different populations and/or individuals for interpreting human history^6,15–19^.

Differently from Y-chromosomal Single Nucleotide Polymorphisms (Y-SNPs), which have very low mutability (~2 * 10^−8^ per base/generation^2^) and are mostly used for identifying haplogroups (i.e. more or less large groups of Y-chromosomal haplotypes sharing an exclusive common ancestor), the Y-STRs typically define single haplotypes and exhibit much higher mutation rates (average order of magnitude is ~10^−3^ per locus/generation). In addition, Y-STR mutation rates show remarkable variability, ranging from ~10^−2^ to 10^−4^ per locus/generation depending on the molecular features of each Y-STR locus, such as the length and the structure of the repetitive unit and the range in the total number of repeats^20–22^. Such variability allowed, for instance, the identification of ‘rapid’ Y-STRs, which may be more apt to individual identification, even when considering close relatives, whereas ‘slower’ markers may be more useful for phylogenetic applications^23^.

Up to now Y-STR typing in forensics as well as in molecular anthropology mostly relied on a limited number of markers which were implemented in a few widely used kits, e.g. the Yfiler, that comprises 17 Y-STRs and, more recently, the PowerPlexY23^24^ and YfilerPlus^25^ kits, that expand the basic Yfiler profile to 23 and 27 markers, respectively.

Further developments in the forensic field brought the identification of a set of 13 Rapidly Mutating (RM) Y-STRs that are characterized by particularly high mutation rates (median: 1.97 * 10^−2^ per locus/generation^23^). Of these, only a handful is available in the most complete commercial kits, namely PowerPlexY23 and YfilerPlus. Their high mutability makes RM Y-STRs ideal tools for increasing the differentiation of unrelated and especially related males, thus contributing to solve cases of homoplasy (i.e. identical haplotype shared between actually unrelated individuals), which is of fundamental importance particularly for individual (suspect) identification^23^. Besides this, RM Y-STRs have been shown to convey a significant phylogenetic signal, which may help to correctly classify individuals belonging to haplogroups characterized by strong demographic expansion and resemblance of Y-STR profiles (such as the widespread Eurasian R-M269 lineage^26–28^).

Indeed, Y-STR mutation rates are key parameters for determining the time of the most recent common ancestor (TMRCA) of distantly related individuals. Available direct estimates of Y-STR mutation rates are mostly based on father-son pairs and/or genealogically related pairs. Different approaches were proposed by Zhivotovsky *et al*.^29^, whose ‘evolutionary rate’ is based on population data and calibrated against well-known historical events, and more recently by Willems *et al*.^30^, who inferred Y-STR mutation rates taking advantage of complete sequencing data. Genealogical and father-son rates were shown to be quite similar to each other – even though some discrepancies were observed for RM Y-STRs – while ‘evolutionary’ rates are significantly slower^28^. A potential complication – which may at least partially explain these differences between direct estimates and ‘evolutionary’ rates – is Y-STRs tendency to homoplasy, i.e. the fact that identical haplotypes may not be the result of a recent shared paternal ancestor, but to the cumulative effect of backmutations^20,31^. However, it has been suggested that the effects of homoplasy are negligible at the time-scale of forensic studies and genealogy-based research^28^, as well as for molecular anthropology studies aimed at reconstructing the most recent events of population history.

The first aim of this study is to provide a wide set of Y-STR mutation rates in the genealogical time-frame for a set of Italian samples. Accordingly, we base our research on deep-rooting pedigrees from Italy and compare our results with those from previous studies that used a direct-count approach (i.e. genealogies and father-son comparisons). We also take advantage of the estimated mutation rates for checking the performance of the RM Y-STRs set and of the most frequently used commercial kits (Yfiler, PowerPlexY23, YfilerPlus). Our second aim is to understand how Y-STRs behave as predictors of the Time of the Most Recent Common Ancestor (TMRCA) in genealogical applications. To do this, we explore the relationship between genealogy depth based on archived records and the corresponding number of Y-STR mutations. As a methodological framework, we implemented the Walsh method^32^ within an R script.

## Results

### Detection of non-paternity events

In this study, we use the term ‘meioses’ for indicating the total number of meiotic events included within a given genealogy. Instead, the term ‘generations’ is used to indicate the average number of meioses separating a pair of related individuals from their common ancestor, which corresponds to the total number of meioses divided by two.

Our dataset comprises 135 individuals forming 63 paternally related pairs and three trios (see Materials and Methods for details). All these samples were typed for 47 Y-STRs loci, including loci from all the most frequently used commercial kits, loci commonly used in genetic genealogy and the full RM Y-STRs panel. In addition, the Y-chromosomal haplogroup information was determined by testing 166 Y-SNPs and the corresponding haplogroup frequencies are reported in Supplementary Table S1 (Supplementary Information). Of the considered pairs/trios, seven pairs and one trio were detected as potentially including non-paternity events. Namely, seven of these cases showed discrepant sub-haplogroup affiliations, while the remaining one exhibited an outlier haplotype (Grubbs test, pval = 0.039). Further runs of the Grubbs test after having removed this haplotype yielded non-significant results (pval = 0.20), thus rejecting the presence of other outliers. Accordingly, the above mentioned seven pairs were excluded from downstream analyses, while the trio was reduced to a pair after removing the non-matching haplotype. This leads to a final set of 57 pairs and two trios (for a total of 120 individuals).

### Calculation of mutation rates

The total number of meioses encompassed in the 59 retained genealogies is 718, while the total number of observed mutations is 248, the vast majority of them (229, i.e. 92.34%) being single-step mutations. Potential multi-step cases are mostly represented by two-step mutations (14), while only a handful of them shows higher numbers of steps (3 three-step and 2 four-step cases). More details on the observed mutations are available in Supplementary Table S2 (Supplementary Information).

Since Y-STR information for 43 additional paternal lineages sampled in the same geographic area of the present study (Emilia-Romagna) was available^9,28^, these data were included in calculations of mutation rates. In fact, that way we could expand the dataset and increase the accuracy of our estimates, at the same time avoiding possible biases related to the geographic origins of the samples (which could imply significantly different haplogroups composition and average generation times, both of them potentially influencing mutation rates^21^). In particular, calculations of mutation rates for STR loci corresponding to Yfiler and RM panels comprise additional information from 29 paternal lineages^9,28^ encompassing 448 further meioses (for a total of 1166), while estimates for DYS481, DYS533, DYS570, DYS576, DYS549, DYS643 (i.e. PowerPlexY23 loci not included in the Yfiler) include additional information from 14 paternal genealogies^28^ corresponding to 92 added generations (for a total of 810). In this study, multi-copy Y-STRs were considered as single loci, calculating their mutation rates as the total number of observed mutations in all STR copies divided by the number of meioses. This was done for practical reasons, since multi-copy STRs may have a variable number of copies, which would complicate the calculation of the ‘average’ mutation rate. Finally, for RM multi-copy loci DYF399S1 and DYF403S1a, one pedigree each was affected by ambiguities in the mutation counts. Since all other pedigrees allowed straightforward counts, we decided to keep these loci and calculated mutation rates after having excluded the two ambiguous cases. This leaded to a total of 1149 meioses for DYF399S1 and 1152 meioses for DYF403S1a.

Mutation rates for all the considered Y-STRs are reported in Table 1. For eight STRs (DYS438, DYS643, DYS459, DYS607, DYS455, DYS454, YCAII and DYS388), no mutations were observed in our dataset. As for the remaining loci, 12 Y-STRs feature rates in the order of magnitude of 10^−2^ per locus/meiosis and are all included in the RM panel (with the exception of DYS724 and DYS464). As expected, six of them (including the four most rapid ones) are multi-copy STRs. All the other 27 considered Y-STRs range in the order of magnitude of 10^−3^ per locus/meiosis, the slowest ones (excluding those with zero mutations) being mostly comprised in the Yfiler panel. It is noteworthy that one of these slow STRs (DYS526A) has actually been included in the RM panel. Such inclusion probably originated from the fact that Y-STRs DYS526A and B were considered as a single locus when the RM panel was firstly defined^20^.

**Table 1:** Typed Y-STRs and estimated mutation rates.

Y-STR	Panel	Number of Mutations		Meioses	Mutation rates (*10-3)						Ref. Values	
		Single	Multi		Val (Single)	CI (95%)		Val (Multi)	CI (95%)		FS	GP
DYS19	Yf/PP/YfP/L	3	3	1166	2.57	0.00	6.00	2.57	0.00	6.00	3.99	2.26
DYS389I	Yf/PP/YfP/L	3	3	1166	2.57	0.00	6.00	2.57	0.00	6.00	5.14	4.24
DYS389II-I	Yf/PP/YfP/L	3	3	1166	2.57	0.00	6.00	2.57	0.00	6.00	3.44	4.80
DYS390	Yf/PP/YfP/L	5	5	1166	4.29	0.86	8.58	4.29	0.86	8.58	1.14	2.54
DYS391	Yf/PP/YfP/L	2	2	1166	1.72	0.00	4.29	1.72	0.00	4.29	2.84	4.52
DYS392	Yf/PP/YfP/L	3	6	1166	2.57	0.00	6.00	5.15	1.72	9.43	0.58	1.13
DYS393	Yf/PP/YfP/L	3	3	1166	2.57	0.00	6.00	2.57	0.00	6.00	1.71	1.13
DYS385 (a-b)	Yf/PP/YfP/L	8	8	1166	6.86	2.57	12.01	6.86	2.57	12.01	5.33	5.37
DYS437	Yf/PP/YfP/L	4	4	1166	3.43	0.86	6.86	3.43	0.86	6.86	1.14	3.11
DYS438	Yf/PP/YfP/L	0	0	1166	—	—	—	—	—	—	0.57	0.28
DYS439	Yf/PP/YfP/L	7	9	1166	6.00	1.72	11.15	7.72	3.43	12.86	3.46	3.67
DYS448	Yf/PP/YfP/L	2	2	1166	1.72	0.00	4.29	1.72	0.00	4.29	0.00	1.13
DYS456	Yf/PP/YfP/L	3	3	1166	2.57	0.00	6.00	2.57	0.00	6.00	4.55	7.91
DYS458	Yf/PP/YfP/L	12	13	1166	10.29	5.15	16.30	11.15	5.15	17.15	7.97	9.04
DYS635	Yf/PP/YfP/L	2	2	1166	1.72	0.00	4.29	1.72	0.00	4.29	3.46	5.65
GATA H4.1	Yf/PP/YfP/L	3	3	1166	2.57	0.00	6.00	2.57	0.00	6.00	2.85	1.98
DYS481	PP/YfP/L	3	3	810	3.70	0.00	8.64	3.70	0.00	8.64	4.59	6.22
DYS533	PP/YfP/L	3	3	810	3.70	0.00	8.64	3.70	0.00	8.64	4.62	3.96
DYS570	PP/YfP/RM/L	9	9	1166	7.72	3.43	12.86	7.72	3.43	12.86	11.92	9.89
DYS576	PP/YfP/RM/L	10	11	1166	8.58	3.43	14.58	9.43	4.29	15.44	13.90	7.91
DYS549	PP/L	8	8	810	9.88	3.70	17.28	9.88	3.70	17.28	4.16	4.52
DYS643	PP/L	0	0	810	—	—	—	—	—	—	1.13	1.13
DYS460	YfP/L	1	1	718	1.39	0.00	4.18	1.39	0.00	4.18	5.82	4.52
DYS518	YfP/RM/L	18	18	1166	15.44	8.58	23.16	15.44	8.58	23.16	17.99	18.65
DYS627	YfP/RM/L	17	19	1166	14.58	7.72	21.44	16.30	9.43	24.01	11.89	15.55
DYF387S1	YfP/RM/L	13	13	1166	11.15	5.15	17.15	11.15	5.15	17.15	15.52	15.26
DYS449	YfP/RM/L	9	9	1166	7.72	3.43	12.86	7.72	3.43	12.86	11.75	11.59
DYS459 (a-b)	L	0	0	718	—	—	—	—	—	—	2.30	2.54
DYS724 (a-b)	L	25	29	718	34.82	22.28	48.75	40.39	26.46	55.71	—	29.11
DYS607	L	0	0	718	—	—	—	—	—	—	—	2.54
DYS455	L	0	0	718	—	—	—	—	—	—	0.00	1.41
DYS426	L	1	1	718	1.39	0.00	4.18	1.39	0.00	4.18	0.00	1.13
DYS454	L	0	0	718	—	—	—	—	—	—	0.00	0.28
DYS447	L	3	3	718	4.18	0.00	9.75	4.18	0.00	9.75	1.74	3.67
DYS442	L	2	2	718	2.79	0.00	6.96	2.79	0.00	6.96	9.35	4.80
DYS464 (a-b-c-d)	L	17	23	718	23.68	13.93	34.82	32.03	19.50	45.96	27.51	—
YCAII (a-b)	L	0	0	718	—	—	—	—	—	—	—	1.13
DYS388	L	0	0	718	—	—	—	—	—	—	0.00	0.28
DYF399S1	RM	70	74	1149	60.92	47.87	74.85	64.40	50.48	79.20	77.48	—
DYS526A	RM	1	1	1166	0.86	0.00	2.57	0.86	0.00	2.57	2.33	—
DYS626	RM	9	10	1166	7.72	3.43	12.86	8.58	3.43	14.58	11.84	—
DYS526B	RM	5	6	1166	4.29	0.86	8.58	5.15	1.72	9.43	12.11*	—
DYS612	RM	16	19	1166	13.72	7.72	20.58	16.30	9.43	24.01	14.15	—
DYS547	RM	15	16	1166	12.86	6.86	19.73	13.72	7.72	20.58	23.23	—
DYF404S1	RM	14	17	1166	12.01	6.00	18.87	14.58	7.72	21.44	12.08	—
DYF403S1a	RM	29	29	1152	25.17	16.49	34.72	25.17	16.49	34.72	30.59	—
DYF403S1b	RM	4	5	1166	3.43	0.86	6.86	4.29	0.86	8.58	11.41*	—
**Panel**												
Yfiler (Yf)		63	69	18656	3.38	2.57	4.23	3.70	2.84	4.61	3.01	3.67
PowerPlex Y23 (PP)		96	103	24228	3.96	3.18	4.79	4.25	3.47	5.08	3.95	4.20
Yfiler Plus (YfP)		146	155	27990	5.22	4.39	6.07	5.54	4.68	6.43	5.74	6.09
Leuven (L)		202	221	37508	5.39	4.67	6.13	5.89	5.12	6.69	—	5.54^
Rapidly Mutating (RM)		239	256	17459	13.69	11.97	15.46	14.66	12.89	16.50	18.80**	—
All		365	398	47971	7.61	6.84	8.40	8.30	7.50	9.11	—	—

Locus-specific and panel-wise estimates (Val) along with the corresponding 95% confidence intervals (CI) are calculated both with all mutations as single events (Single) and with multi-step mutations as sum of independent events (Multi). Reference values extracted from the literature are also included, with FS = Father-Son pairs^20^ and GP = Genealogical Pairs^21^. Significant comparisons (Fisher tests) are highlighted in bold, with *p < 0.05 and **p < 0.005.

**Figure 1:**
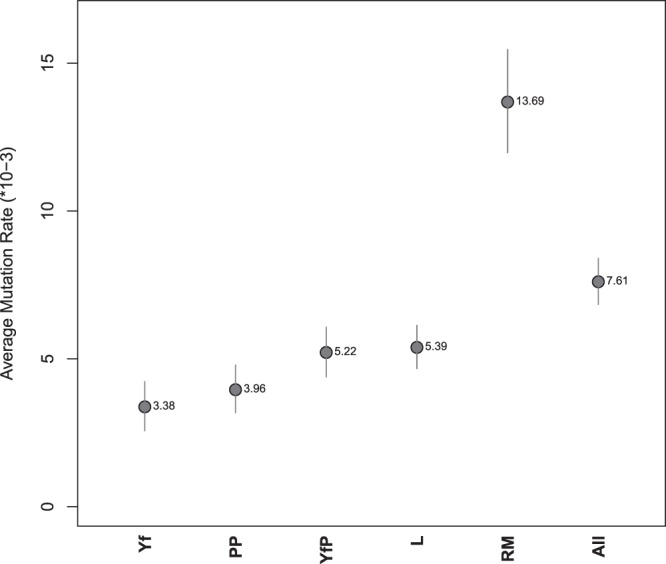
Overall mutation rates and 95% confidence intervals for the considered Y-STR sets (abbreviations as in Table 1).

At a panel-wise level (Table 1, Fig. 1) we observe that the Yfiler set (Yf) exhibits the lowest average mutation rate. Consistently with previous studies^28,33^, the relatively slow mutation rates of the loci included limit the power of Yfiler to completely resolve individual differentiation among related males. By contrast, the Rapidly Mutating Y-STRs set (RM) is by far the ‘fastest’ one, thus confirming its potential for detecting differences even between related individuals^23^. PowerPlexY23 (PP) average mutation rate is similar to that of Yf, while YfilerPlus (YfP) and in-house Leuven (L) panels exhibit higher rates, which however remain quite distant from that of RM.

### Mutation rates in meioses bins

**Figure 2:**
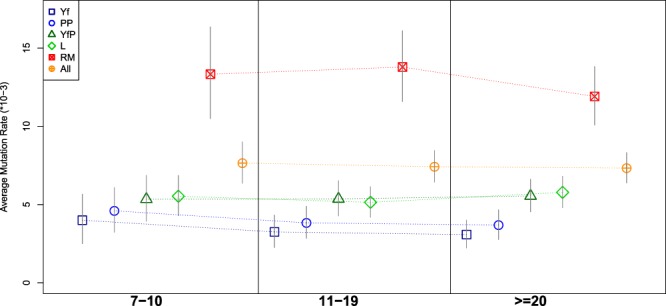
Diachronic changes of overall mutation rates for the considered Y-STR sets (abbreviations as in Table 1) on three increasing bins of meioses (7–10, 11–19, >19).

In order to check if mutation rates are influenced by changes in temporal depth of genealogies, we organized our data according to pairwise comparisons (within genealogies), including previously published data^9,28^ when overlapping with the Y-STR sets used in this study. In particular, mutation rates were calculated for three bin groups, based on the number of meioses separating pairs of individuals from their common ancestor, i.e. 7–10, 11–19, >19. Obtained results (Fig. 2, Supplementary Table S3, Supplementary Information) show that nor the considered Y-STR panels (Yf, PP, YfP, L, RM) neither the whole dataset (All) exhibit any significant variation between the three temporal cohorts. In fact, the corresponding confidence intervals are highly overlapping, thus suggesting no significant traces of saturation in the considered time frame (i.e. up to ~30 meioses, corresponding to ~15 generations ago).

### Average generation time

Within the considered genealogies, we detected reliable information on the age of the fathers at the time of birth of their sons for 692 meioses and the corresponding total number of years is 23,230. This leads to an estimate of average generation time equal to 33.57 years (with 95% bootstrapped confidence interval comprised between 33.00 and 34.13).

### Relationship between the number of meioses and the number of observed mutations

In theory, we would expect that the higher the number of meioses, the higher the number of observed mutations. Indeed, when modeling the relationship between mutations and time depth with a linear regression model (Table 2), we obtain significant results not only for the whole dataset but also for some of the considered Y-STR panels. Among them, the best-fitting ones are L, which includes the highest number of markers, and RM, which exhibits the highest mutation rates. By contrast, PP and Yf panels show a much lower association with time depth (as represented by their low regression coefficients and R-squared values; Table 2), which however occasionally reaches the significance threshold. Spearman rank correlation values fully confirmed the above results (Table 2).

**Table 2:** Panel-specific and All markers data summary statistics of linear regression models with TMRCA as a function of the number of observed mutations and Spearman rank correlation with the same variables.

Panel	Single/Multi	Nr. Meioses	Regression coefficient	P-value	R-squared		Spearman	
					Multiple	Adjusted	Rho	P-value
Yfiler	Single	1941	0.04688	4.850E-04	0.09174	0.08458	0.3119116	3.202E-04
	Multi		0.06083	7.746E-05	0.11610	0.10920	0.3345687	1.065E-04
PP23	Single	870	0.05334	3.833E-02	0.05904	0.04578	0.2725257	1.967E-02
	Multi		0.07743	6.171E-03	0.10090	0.08824	0.3092618	7.760E-03
YfilerPlus	Single	761	0.10090	1.144E-03	0.16040	0.14660	0.4202341	6.059E-04
	Multi		0.13253	8.568E-05	0.22510	0.21240	0.4675362	1.118E-04
RM	Single	1941	0.15780	1.918E-12	0.32410	0.31880	0.5891791	2.062E-13
	Multi		0.15760	3.798E-10	0.26650	0.26070	0.5365281	5.616E-11
Leuven	Single	761	0.19030	5.564E-06	0.28890	0.27720	0.4963118	3.522E-05
	Multi		0.26460	3.761E-07	0.34720	0.33650	0.5458658	3.709E-06
All markers	Single	761	0.32620	1.918E-09	0.44880	0.43980	0.5763884	7.675E-07
	Multi		0.39730	2.584E-09	0.44350	0.43440	0.5713817	1.005E-06

Calculations were performed considering all mutations as single events (Single) and multi-step mutations as sum of independent events (Multi). Panel abbreviations as in Table 1.

### Walsh method

In this study, we implemented both the IAM and SMM variants of the Walsh^32^ method (see Methods for details) and applied them to our data. Analyses were limited to the whole dataset (All) and to the Y-STR panels that showed a significant and strong association between the number of observed mutations and the number of generations, i.e. RM and L.

Overall, our results (Table 3, Supplementary Table S4, Supplementary Information) reveal a good association between the TMRCAs estimated with the Walsh method and the genealogy-documented ones. Among the considered Y-STR panels, the best fit was obtained with the RM set. As expected, an even higher fit was observed for the complete dataset (All). In addition, All-based estimates yield tighter CIs combining the best match with the highest precision.

**Table 3:** Performance of the Walsh^32^ procedure as the percentage of observed TMRCA values falling within the estimated confidence intervals using the whole dataset (All data) along with Rapidly Mutating (RM) and Leuven (L) panels and two different mutation models (IAM = Infinite Alleles Model; SMM = Stepwise Mutation Model).

Panel	Model	% TMRCA within estimated CIs
RM	IAM	96.72%
RM	SMM	93.44%
L	IAM	91.80%
L	SMM	88.52%
All data	IAM	96.72%
All data	SMM	91.80%

Both All and RM best fits were associated to the IAM model (96.72% of observed TMRCA values falling within CIs of the estimated ones). By contrast, the least effective match was observed with the Leuven set and the SMM model (88.52%). Furthermore, in all cases the SMM-based estimates performed worse than the corresponding IAM ones. This may be a consequence of the fact that the strict SMM does not take into account the possibility of multi-step mutation events, while considering changes in allele states as the increase or decrease by a single repeat unit at a time. Therefore, the corresponding estimates tend to be higher than the IAM ones.

All cases that were previously identified as potential non-paternity events yield estimates that are significantly higher than the documented number of generations, with all the considered panels and mutation models (Supplementary Table S4, Supplementary Information). By contrast, all other observed discrepancies involve only one or few panels/models. Therefore, we suggest that the above procedure may be used as reliable tool for identifying non-paternity events with Y-STR data.

As previously observed by Walsh^32^, the posterior distributions of TMRCAs are skewed to the right, with mean > median > mode (Supplementary Fig. S1, Supplementary Table S4, Supplementary Information). We asked ourselves which of these three summary statistics best match with the observed values. As a first overview, we calculated the deviation between estimated and observed values by weighting them for the corresponding degrees of freedom. We notice that, while almost all summary statistics tend to overestimate the actual values, in general IAM estimates exhibit lower deviation than SMM ones. As expected, deviations follow the same trend of the considered summary statistics, with the highest values for means and the lowest for modes. Therefore, modes of the posterior distributions seem to best approximate the observed values.

When regressing observed values (y) against expected ones (x), all the considered statistics/panels/models yielded highly significant results (Table 4), thus confirming the strong association between our estimates and the corresponding observed values, with the highest regression coefficient resulting for IAM estimates. However, all regression lines do not perfectly match the identity line, suggesting that neither IAM nor SMM, at least as modeled by Walsh^32^, fit the actual STR mutation processes. It is worth mentioning that, if regressions are set through the origin (that is conditioning the model to the fact that estimated TMRCA = 0 should correspond to observed TMRCA = 0), all regression coefficients increase, in one case (All dataset, IAM model) being in agreement with the one of the identity line (Supplementary Fig. S2, Supplementary Information). These results suggest that, even in the best case, both models (IAM, SMM) tend to underestimate TMRCAs when the observed values are relatively low (<6) and to overestimate them for higher numbers of generations.

**Table 4:** Deviation and parameters of both normal regression models and regressions through the origin, with observed TMRCA values as a function of the expected ones.

Panel	Model	Statistic	Deviation	Regression With Intercept						No Intercept		
				Beta	CI_2.5%	CI_97.5	Pval	R^2^_M	R^2^_A	Beta	CI_2.5%	CI_97.5%
RM	SMM	MEAN	4.73	0.25	0.16	0.33	1.28E-07	0.38	0.37	0.47	0.42	0.53
RM	SMM	MEDIAN	3.34	0.27	0.18	0.36	1.17E-07	0.38	0.37	0.53	0.46	0.60
RM	SMM	D.MODE	1.13	0.31	0.21	0.40	6.59E-08	0.39	0.38	0.64	0.55	0.73
RM	IAM	MEAN	2.87	0.35	0.23	0.46	6.81E-08	0.39	0.38	0.60	0.54	0.67
RM	IAM	MEDIAN	1.98	0.35	0.24	0.46	7.00E-08	0.39	0.38	0.65	0.58	0.72
RM	IAM	D.MODE	0.31	0.36	0.24	0.47	4.61E-08	0.40	0.39	0.74	0.64	0.84
L	SMM	MEAN	4.24	0.21	0.13	0.28	1.38E-06	0.33	0.32	0.46	0.39	0.52
L	SMM	MEDIAN	3.25	0.21	0.13	0.29	1.38E-06	0.33	0.32	0.48	0.41	0.56
L	SMM	D.MODE	1.31	0.22	0.14	0.31	1.01E-06	0.34	0.32	0.54	0.45	0.64
L	IAM	MEAN	2.72	0.26	0.14	0.38	7.46E-05	0.24	0.22	0.59	0.51	0.66
L	IAM	MEDIAN	1.88	0.26	0.14	0.38	7.77E-05	0.23	0.22	0.62	0.53	0.71
L	IAM	D.MODE	0.13	0.27	0.15	0.40	4.76E-05	0.25	0.23	0.71	0.59	0.83
All data	SMM	MEAN	3.07	0.30	0.21	0.39	1.63E-08	0.42	0.41	0.56	0.49	0.62
All data	SMM	MEDIAN	2.46	0.30	0.21	0.40	1.57E-08	0.42	0.41	0.58	0.51	0.66
All data	SMM	D.MODE	1.38	0.31	0.22	0.40	1.13E-08	0.43	0.42	0.63	0.55	0.71
All data	IAM	MEAN	1.11	0.47	0.31	0.62	8.43E-08	0.39	0.38	0.77	0.69	0.85
All data	IAM	MEDIAN	0.61	0.47	0.32	0.62	8.23E-08	0.39	0.38	0.81	0.73	0.90
All data	IAM	D.MODE	−0.40	0.46	0.31	0.62	1.28E-07	0.38	0.37	0.90	0.79	1.00

Calculations were performed for the complete dataset (All data) and the two best-fitting panels (RM and L; abbreviations as in Table 1) and three summary statistics (median, mean and mode). Regression coefficients (Beta) and their 95% confidence intervals are reported for all models, while p-values and R-squared statistics (M = Multiple, A = Adjusted) only for regressions with intercept.

## Discussion

Y-chromosomal studies still play a relevant role in current human genomics research. Moreover, they are of great interest in interdisciplinary research, next to a world-wide audience of citizen science practitioners^4^. Mutation rates are key parameters for understanding how Y-STRs impact the variability of human Y-chromosome and, consequently, for their use in forensic sciences, genetic genealogy, human population genetics and molecular anthropology. For instance, knowledge of mutation rates is of the greatest importance for designing and predicting the discriminatory power of a given Y-STR panel^20^. Similarly, estimating the TMRCA of a pair or a group of related Y-STR haplotypes relies on their mutation rates, as well as on the average generation time^2^.

In this study, we provide for the first time a wide set of Y-STR data sampled from Italian genealogical pairs, i.e. pairs of individuals who share a documented, common paternal ancestor. The above dataset includes markers from all the Y-STRs panels most commonly used in forensics and molecular anthropology (Yfiler, PowerPlexY23, YfilerPlus), additionally embracing all markers from the Rapidly Mutating panel (RM) and a further set of Y-STR loci almost exclusively used for genetic genealogical approaches, among them the multi-copy marker DYS464. This locus, which has been suggested as a particularly informative one in forensics^34^, indeed revealed a remarkable variability in our data (overall mutation rate: 2.368 * 10^−2^ per locus/meiosis).

We used this information for estimating mutation rates and exploring their relationship with TMRCA, in addition testing the performance of both classic mutation models – the Infinite Alleles Model (IAM) and the Stepwise Mutation Model (SMM) – that were here developed in the Bayesian framework proposed by Walsh^32^.

On the whole, our data comprise 47 Y-STRs and 166 Y-SNPs typed in a set of 135 individuals from 66 paternally related namesakes, which, for some markers, were augmented up to 234 individuals and 95 paternal genealogies by including previously published paternally-related individuals from the same geographic area^9,28^, so as to maximize the accuracy and the representativeness of our dataset. After having excluded those markers for which no mutations were observed, the calculated mutation rates fall within a range from 10^−2^ to 10^−4^ per locus/meiosis, thus confirming previous estimates^20^.

In particular, locus-specific mutation rates (Table 1) generally agree with those reported in two previous reference papers, i.e. Ballantyne *et al*.^20^ and Claerhout *et al*.^21^. The first one is based on father-son comparisons, while the second used genealogical pairs such as in the present study. For reasons of simplicity, all comparisons were performed considering multi-step mutations as single events (and recalculating comparison mutation rates if necessary). Interestingly, those markers that show the highest differences between Ballantyne *et al*.^20^ and our data are mostly included in the RM panel (particularly DYS526B and DYF403S1b; p-values 0.039 and 0.024, respectively, according to Fisher tests). More precisely, our estimates for these markers are significantly lower than those calculated with father-son pairs. This finding is confirmed by panel-wise comparisons (Table 1, Supplementary Fig. S3, Supplementary Information), in which the only significant difference with respect to Ballantyne *et al*.^20^ has been observed for RM Y-STRs, that in fact show a significantly higher value than ours. No significant difference was instead observed when comparing our results with those by Claerhout *et al*.^21^, that share the same genealogy-based approach as well as most of the markers considered in the present study (excepted for nine RM Y-STRs).

The vast majority of mutations observed in our dataset (92.34%) correspond to single-step mutational events, while only a handful of them involve potential multi-step mutations. Compared to Ballantyne *et al*.^20^, who relied on father-son pair comparisons and therefore could identify genuine multi-step events occurring in a single meiosis, their frequency in our dataset is higher (4% and 7.66%, respectively) and more similar to that observed by Claerhout *et al*.^21^ (6.9%). These results would suggest that some of the multi-step events present in our data were actually due to two (or more) independent events that occurred at the same marker during the time frame spanned by the considered paternal lineages. By combining this observation with the fact that the only significant differences with father-son pairs^20^ were observed for RM markers, we conclude that fast-evolving STRs are the most likely candidate for multiple mutations at the same locus. However, the enrichment in multi-step mutations observed in our results compared to Ballantyne *et al*.^20^ do not reach statistical significance (binomial test: pval = 0.19), thus confirming that the impact of pseudo-multi-step events in our results is on the whole negligible.

A particular case of multiple mutations at the same locus is provided by back-mutations, i.e. mutation events that over-ride the previous ones, thus potentially leading to saturation (i.e. apparently identical Y-STR profiles in individuals that do not share a recent common ancestor). If back-mutations affected our data, we would observe lower mutation rates for genealogies with higher number of meioses. Calculation of mutation rates in three classes of genealogical pairs with different time depth (7–10, 11–19, >19; Fig. 2, Supplementary Table S3, Supplementary Information) revealed no significant differences for any of the considered STR sets, including RM, therefore suggesting that not only pseudo multi-step mutations but also saturation did not affect significantly our results. However, we caution that this may not be the case when dealing with larger time depths than those involved in documented genealogies.

Compared to father-son pairs, which can be used essentially to calculate mutation rates, the genealogy-based approach allows further investigations of the relationship between mutations and time. In fact, each of the sampled namesakes can be associated to a specific number of separating meioses as well as to precise birth dates of the involved ancestors. In this respect, we show how our data can be used to get precise estimates of the average generation time. In addition, it is worth noting that the result obtained here (33.57 years, CI: 33.00, 34.13) agrees very well with previous estimates from the same geographic domain (33.38 years^9^, CI: 32.76, 34.00).

Indeed, Y-STR variability – coupled with average generation time estimates – has been widely used to infer the age of groups of haplotypes and even whole Y-chromosomal haplogroups^35–37^. This approach has been recently criticized since the peculiar mutation modality of STRs – characterized by back-mutations and homoplasy – would inevitably bias time estimates^31,38^. However, when considering more restricted time scales, such as genealogical ones, these issues are less likely to significantly affect the calculations. Accordingly, our results suggested that the effects of homoplasy and multiple mutations at the same locus are negligible in our dataset.

Among the considered STR panels, those including a lower number of markers and exhibiting moderate/low mutation rates (Yfiler, PowerPlexY23) revealed poorly-fitting relationships with the number of generations (Table 2), instead yielding non-significant regressions and/or low R-squared values. By contrast, fitting models were obtained not only with the whole dataset (All), but also with panels comprising an higher number of markers or the most rapidly mutating ones (L and RM), and thus yielding a relatively higher number of mutations.

These results seem to suggest that, in theory, it could be possible to estimate the TMRCA of a pair of related haplotypes with a reasonable error, obviously assuming that a sufficient amount of information is provided. In our case (Table 1), with the RM dataset we were able to provide 0.21 mutations per meiosis (15 loci), while the complete dataset (All) yielded 0.38 mutations per meiosis (47 loci). In order to test this possibility, we compared the number of generations to the TMRCA provided by genealogical information (calculated as the number of documented meioses divided by two) with the same values independently estimated from haplotypic Y-STRs data.

In particular, we applied the method proposed by Walsh^32^ for inferring the TMRCA of a pair of given haplotypes using the complete dataset (All) and the best-fitting panels, namely L and RM. The procedure designed by Walsh^32^ has been set in a Bayesian framework and implements two different mutation models, i.e. the Infinite Alleles Model (IAM) and the Stepwise Mutation Model (SMM). The first model (IAM) considers all mutations as unique events – no matter if single or multi-step – while the second one allows for multiple, symmetric one-step mutations, meaning that all multi-step mutations are interpreted as the result of multiple independent events. Therefore, IAM may underestimate the actual number of mutations, while SMM may overestimate it.

In theory, SMM should best model the peculiar STR mutational process, characterized by the increase or the decrease of a single repeat unit at a time. However, our results clearly indicate that the model which best reproduces the observed data is IAM for all the considered sets of markers (Table 3, Supplementary Table S4, Supplementary Information). This result would suggest that, at least in the relatively short time-frame covered by pedigrees, the Infinite Alleles Model, which in theory should be more akin to SNP-like mutation mechanisms, fits better to the observed data than the STR-specific SMM. On the other hand, this observation fully agrees with the aforementioned negligible impact of multiple mutations at the same locus in our dataset. Summing up, SMM tends to overestimate the observed TMRCAs because ‘genuine’ multi-step mutations are not allowed by the model. In addition, SMM produces larger CIs at the considered time scale, hence leading to less precise estimates. Among the considered panels, the best correspondences was again obtained with RM. When using the complete dataset (All), 96.72% of the observed TMRCAs fall within CIs predicted by the IAM, and the best-fitting models (when regressing observed TMRCAs against expected ones) were obtained. Among the considered summary statistics, we particularly observed that the modal value of the posterior distribution (Supplementary Fig. S1, Supplementary Information) is the one that best fits with the observed TMRCA values (Table 4, Supplementary Fig. S2, Supplementary Information).

Our results also suggest that the Walsh procedure allows the identification of potential non-paternity events within genealogical pairs by estimating significantly higher TMRCAs than those documented by genealogical data with all the considered sets of markers and models (Supplementary Table S4 Supplementary Information). In fact, based on our Italian dataset, this condition is met for the seven genealogical pairs showing non-coinciding haplogroups as well as for the outlier haplotype identified using the Grubbs test. This result corroborates previous applications of the Walsh formula (albeit with a less thorough approach) to similar datasets^9,13,39^.

However, expected TMRCAs tend to overestimate observed ones when their number is lower than ~6 generations, while underestimating them for higher values. Thus, TMRCA estimates are more prone to false estimates in deeper time layers, such as those typically considered in population genetics studies. However, we believe that these issues may be addressed by allowing for more complex models than those originally provided by Walsh^32^ and by considering wider datasets.

## Conclusions

In this study, we contributed to the knowledge of Y-STR mutation rates by introducing new genealogy-based estimates from pairs/trios of related individuals sampled in Northern Italy. At the same time we provide a wide set of Y-STR data – including all the most frequently used forensic panels, Rapidly Mutating (RM) Y-STRs and further 11 Y-STR markers – which represents a valuable addition to the already available Italian Y-STR information.

Overall, our results confirm that the genealogy approach allows reliable estimates of mutation rates to be obtained even starting from a relatively restricted number of individuals, also showing that the impact of multiple mutations at the same locus is negligible, at least within the temporal scale usually adopted by forensic and genetic genealogy analyses.

The genealogy approach moreover gives us the opportunity to test the relationship between TMRCAs and mutations. Indeed, we detected a significant association between these variables when using not only the complete dataset but also the Leuven and RM panels, i.e. those marker sets characterized by a high number of markers and high mutation rates, respectively. Accordingly, we suggest that such association may be used to estimate unknown TMRCAs in genetic genealogy, familial searching and forensics. To this end, we tested the procedure proposed by Walsh^32^ for inferring the TMRCA between pairs of haplotypes, and demonstrate that it yields a good performance, especially when adopting the Infinite Alleles Model (IAM), which, at the considered time scale, seems to better approximate the observed mutation patterns than a strict Stepwise Mutation Model (SMM). As expected, the best-fitting TMRCA estimates were obtained when using the whole (All) dataset. However, based on our Italian data, the RM panel is a good compromise between performance and number of typed markers.

Therefore, the Walsh procedure seems a promising tool especially for genetic genealogy and familial searching applications. In perspective, it could be applied also to forensics, as a method for excluding hypotheses of relatedness between two individuals, and to molecular anthropology studies, as a tool for estimating the TMRCA of individuals with more ancient relatedness. However, further testing on wider datasets, as well as the introduction of more parameters into the models – for instance allowing for locus-specific mutation rates and for multi-step events in SMM – are needed to improve the procedure.

## Materials and Methods

### Population sampling

The present study comprises 135 samples belonging to 66 different paternal lineages from two bordering regions of Northern Italy, i.e. Emilia-Romagna and Veneto. For each paternal lineage, a pair of related individuals has been considered, excepted for three cases, which are instead represented by trios of individuals. Each pair/trio of paternally related individuals shares the same surname, except for a few instances where spelling variants of the same surname were found. For ethical reasons, the related participants needed to be separated by at least seven meiotic divisions. The relatedness of the pairs/trios of sampled individuals was proved through comprehensive archival data, namely civil registers, parish registers and notarial acts. DNA samples were collected with buccal swabs in 2016 and 2017 sampling campaigns from adult and healthy male volunteers. Written informed consents were obtained for permission on both DNA analysis and on the scientific publication of anonymized results. The Bioethic Committee of the University of Bologna approved all the procedures. The samples were processed in a linked but anonymized form, and the confidentiality of personal information for each participant to the study was assured. The present study was designed and performed in accordance with relevant guidelines and regulations and according to ethical principles for medical research involving human subjects stated by the WMA Declaration of Helsinki.

In order to increase the dataset and the statistical power of our analyses, we also included available Y-STRs data for 99 further individuals representing 29 additional paternal genealogies, who were sampled in two different locations of Emilia-Romagna^9,28^, therefore matching the same geographic area of the present study.

### Laboratory methods

Whole genome DNA was extracted by means of a salting out protocol modified from Miller^40^ and quantified with the Qubit® dsDNA HS Assay Kit (Life Technologies, Carlsbad, CA, USA).

Newly collected samples were genotyped for 27 Y-STR loci, including those in the commercially available Yfiler™, Yfiler® Plus (Thermo-Fisher Scientific, USA) and PowerPlex® Y23 System (Promega, Madison, WI, USA) genotyping kits: DYF387S1a/b, DYS19, DYS385a/b, DYS389I, DYS389II, DYS390, DYS391, DYS392, DYS393, DYS437, DYS438, DYS439, DYS448, DYS449, DYS456, DYS458, DYS460, DYS481, DYS518, DYS533, DYS549, DYS570, DYS576, DYS627, DYS635, DYS643 and GATA H4.1. In addition, 11 other Y-STR loci which are often used in genetic genealogical applications were also included, i.e. DYS388, DYS426, DYS442, DYS447, DYS454, DYS455, DYS459a/b, DYS464a/b/c/d, DYS607, DYS724a/b and YCAIIa/b.

DNA was amplified using 6.25 µl Qiagen® Multiplex PCR Kit (Qiagen), 1.75 µl AmpSolutionTM Reagent (Promega, Madison, WI, USA), 2.5 µl primer mix and 3 µl of DNA extract. PCR conditions were set as described by Jacobs *et al*.^41^ and Claerhout *et al*.^21^. The amplified DNA samples were purified with the BigDye XTerminator® Purification Kit (Applied Biosystems). The purified DNA samples were plated out in Hi-DiTM Formamide on an ABI PRISM 3130 XL Genetic Analyzer with POP7 and a 50 cm capillary (Applied Biosystems) using GeneScan™ 500 LIZ™ Dye Size Standard (Applied Biosystems) as a size standard. Fragment length analysis of the 46 Y-STRs was done using GeneMapper® v3.2.1 (Applied Biosystems).

All the samples were also typed for the full set of 15 Rapidly Mutating (RM) Y-STRs (we considered DYF403S1a/b and DYS526A/B as separated loci) by using three multiplex PCR assays as described in Robino *et al*.^42^. As six RM loci (DYS570, DYS576, DYS518, DYS627, DYF387S1, DYS449) were typed with both procedures, we could confirm their concordance after comparing the obtained alleles.

Next, all individuals were additionally genotyped by using multiplex SNaPshot mini-sequencing assays (Thermo Fisher Scientific, Waltham, MA, USA) as described in Claerhout *et al*.^21^. A total of 166 Y-SNPs were divided in 23 MP kits to confirm the haplogroup and to define the sub-haplogroup of each DNA sample (Supplementary Text S1, Supplementary Information). For amplification, 2 µl of DNA extract was added to 23 µl of amplification mix containing 12.5 µl 2x QIAGEN Multiplex PCR Master Mix, 0.5 µl of Nuclease-Free H2O (Promega, Madison, WI, USA), 5 µl of 5X AmpSolution™ Reagent, and 5 µl of the relevant multiplex primer mix. PCR conditions were: 15 minutes at 95 °C, 30 cycles of 30 seconds at 94 °C, 30 seconds at 55 °C and 90 seconds at 72 °C followed by a final extension of 10 minutes at 72 °C. Analysis of the amplified DNA samples was done with SNaPshot® Multiplex System for SNP genotyping (Applied Biosystems) as described previously^43–45^.

### Statistical methods

The pedigree-based dataset gave the opportunity to confirm the biological kinship between the namesakes through Y-chromosomal comparison^46^. In order to detect potential non-paternity events, we adopted the following procedure: (a) for each pair/trio, we compared the sub-haplogroup affiliation of the respective individuals and considered haplogroup discrepancies as evidence of a non-paternity event; (b) then, we searched for outlier genealogies among those pairs/trios showing sub-haplogroup concordance, i.e. pedigrees revealing an outlier number of mutations compared to the number of meioses separating the individuals^28^. Outlier genealogies were identified by iteratively applying the Grubbs test to the mutations/meioses ratio calculated for each pair/trio (function *grubbs.test*, outlier package, R software^47,48^). All genealogies identified with the above-mentioned procedure were excluded from mutation rates estimates.

Y-STR mutation rates for all the considered markers were estimated by direct counting. For each pair/trio of related individuals, we counted the number of meioses and the number of mutations separating the corresponding haplotypes. As for multi-step mutations – i.e. gain/loss of more than one repeat at a time – we considered them both as single events (i.e. genuine multi-step mutations) and as a sum of independent events (each of them being a single-step). Accordingly, locus-specific mutation rates were calculated for both mutation counts (i.e. multi-step mutations as either single or multiple events) by dividing the total number of mutations for the total number of meioses. The individual mutation rate for the locus DYS389II was estimated by subtracting the DYS389I from the DYS389II so as to avoid double count of mutations. In order to allow maximal resolution and to compare our results with literature ones, multi-copy STRs were generally considered as independent loci in the same way as a unique single locus, by assuming that – given the relatively short time frame covered by documented paternal genealogies – identical configurations do correspond to identical haplotypes. However, when ambiguities in the determination of the number of mutations arose, the corresponding genealogical pairs were excluded from calculations.

In addition, we calculated panel-specific mutation rates by averaging the corresponding locus-specific mutation rates for the number of loci. The wide set of markers typed for this study (All) allowed us to perform analyses for Y-STRs corresponding to the following commercially available panels: Yfiler (Yf), PowerPlex23 (PP) and YfilerPlus (YfP). We also included the Rapidly Mutating Y-STRs panel (RM) and the whole in-house panel from Claerhout *et al*.^21^ (Leuven, L).

Confidence intervals (CIs) were calculated by estimating 2.5% and 97.5% quantiles of a binomial distribution with n = number of meioses and p = number of observed mutations / number of meioses (as implemented in the R function *qbinom*^48^).

Diachronic variations in mutation rates were assessed by grouping genealogical pairs in three bin classes based on the number of meioses separating the corresponding individuals, namely 7–10, 11–19, >19. These intervals were selected in order to have comparable numbers of meioses in the three groups. When genealogies encompassed more than two individuals, we considered all the possible pairs within the pedigree itself. Finally, mutation rates were calculated by repeating the same above-mentioned procedure.

Average generation time was obtained starting from all the available ages and/or years of birth of the individuals included in the paternal genealogies. For each genealogy, we counted the number of years and the number of meioses encompassed between its root and the leaves. Finally, we divided the total number of years for the total number of meioses. Confidence Intervals (95%) were calculated by randomly sampling branches of the pedigrees with a bootstrapping procedure (1000 replications).

The relationship between mutations and time was further explored by fitting linear regression models where the explicative variable (x) is the number of meioses and the response variable (y) is the number of observed mutations. Various models were fitted and tested considering different STR sets. In addition, we calculated Spearman’s rank correlation using the same variables. Calculations were based on the R functions *lm* and *cor.test* respectively^48^.

Finally, we estimated TMRCA (Time to the Most Recent Common Ancestor) for each of the considered pairs (trios were decomposed in pairs of samples) by adopting the method proposed by Walsh^32^. The original equation by Walsh was set in a Bayesian framework and used to generate posterior distributions of TMRCA based on the number of observed mutations for each locus and given a specific model. As for Y-STRs, Walsh^32^ particularly considered the following models: 1) the Infinite Alleles Model (IAM) and 2) the Stepwise Mutation Model (SMM). According to IAM, only a single mutation may occur at a given marker, while SMM allows for multiple, symmetric single-step mutations. The IAM is generally associated with Unique Event Polymorphisms, such as SNPs and Indels; however in a relatively short time-scale, such as the one covered by genealogical information, IAM may be considered as a good approximation also for STRs. Instead, the SMM is specifically designed upon the mutational mechanism and the relatively high mutation rates of STRs. In this study, we implemented both the IAM and SMM variants of the Walsh method and applied them to our data. Analyses were based on the Y-STR sets that showed a significant and strong association between the number of observed mutations and the number of generations.

Comparisons between observed and estimated TMRCAs were firstly performed by checking the congruence between the estimated CIs and the observed values. Then, we modeled linear regressions with observed values on the y-axis and estimated ones on the x-axis^49^. The Walsh procedure was implemented with an R script, which is made available in the Supplementary Materials (Supplementary Text S2, Supplementary Information).

## Supplementary information

Supplementary information

Supplementary Tables S1-S4

Walsh Rscript

## Data Availability

The Y-STR data of all samples are available on the YHRD database (www.yhrd.org) with accession numbers  YA004601.
