# Supplementary material for: Estimating Y-Str Mutation Rates and Tmrca Through Deep-Rooting Italian Pedigrees
Source: Sci Rep. 2019 Jun 21;9:9032. doi: 10.1038/s41598-019-45398-3 (PMC6588691; doi:10.1038/s41598-019-45398-3)
Supplement: Supplementary file 1 — Supplementary information [file 41598_2019_45398_MOESM1_ESM.pdf]

ESTIMATING Y-STR MUTATION RATES AND TMRCA THROUGH DEEP-ROOTING ITALIAN PEDIGREES

Alessio Boattini[1,^,*], Stefania Sarno[1,^], Alessandra M. Mazzarisi[1], Cinzia Viroli[2], Sara De Fanti[1], Carla Bini[3], Maarten H.D. Larmuseau[4,5], Susi Pelotti[3,#], Donata Luiselli[6,#]

[1] Dipartimento di Scienze Biologiche, Geologiche e Ambientali (BiGeA), Università di Bologna, 40126, Bologna, Italy

[2] Dipartimento di Scienze Statistiche "Paolo Fortunati", Università di Bologna, 40126, Bologna, Italy

[3] Dipartimento di Scienze Mediche e Chirurgiche, Università di Bologna, 40126, Bologna, Italy

[4] Laboratory of Forensic Genetics and Molecular Archaeology, Forensic Biomedical Sciences, KU Leuven, B-3000, Leuven, Belgium

[5] Laboratory of Socioecology and Social Evolution, Department of Biology, KU Leuven, B-3000, Leuven, Belgium

[6] Dipartimento di Beni Culturali, Università di Bologna, 48121, Ravenna, Italy

^ These authors contribute equally to this work

# These authors share senior authorship

*Corresponding author: Alessio Boattini, Department of Biological, Geological and Environmental Sciences, University of Bologna, Via Selmi 3, 40126, Bologna, Italy.

E-mail address: alessio.boattini2@unibo.it

Supplementary Information

Includes Supplementary Figures S1-S3, Supplementary Tables S1-S4, Supplementary Text S1-S2.

Supplementary Figures

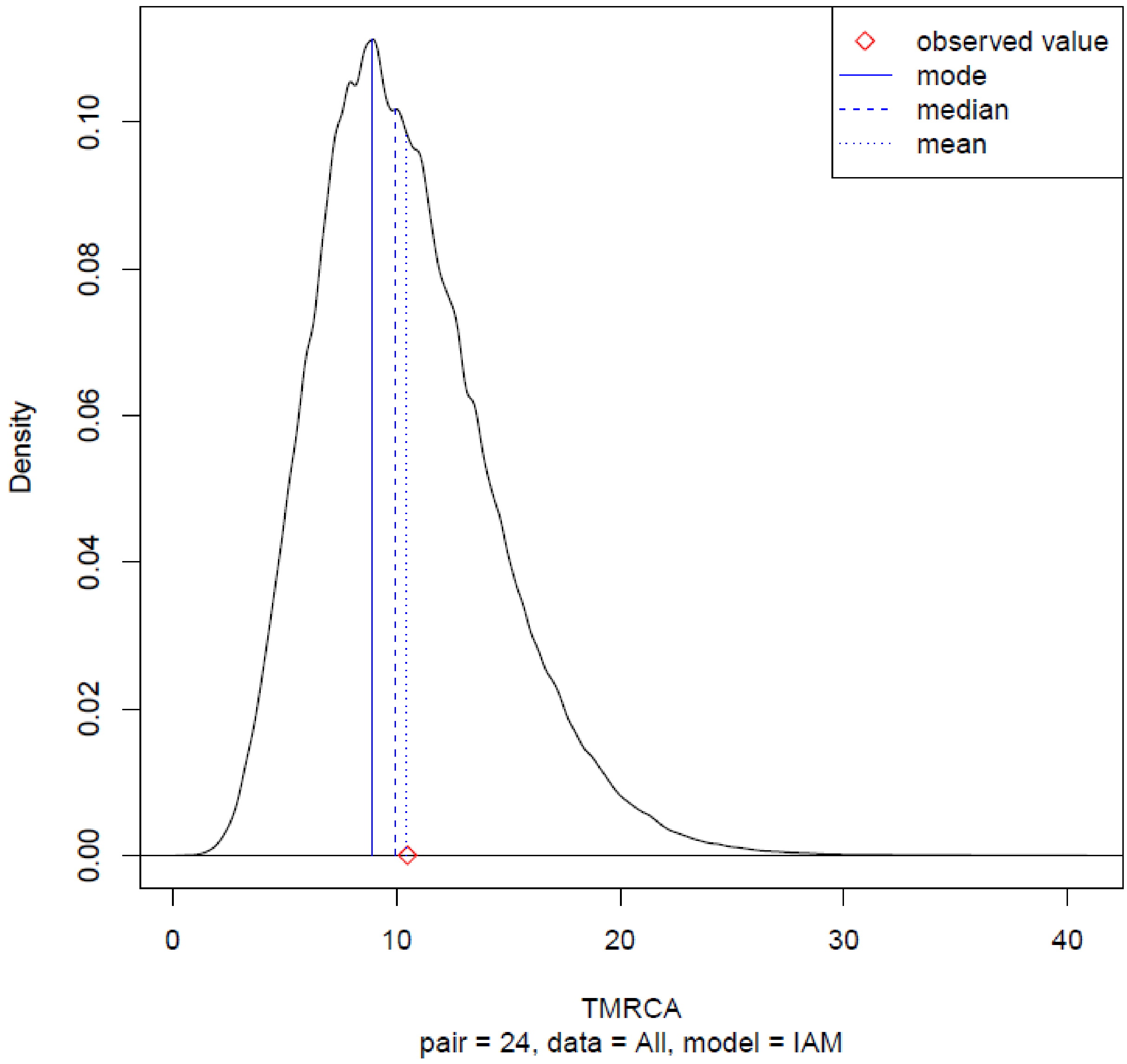

Supplementary Figure S1. Example of a posterior distribution of the TMRCA estimated with the Walsh[32] procedure. The distribution is clearly skewed with mean > median > mode.

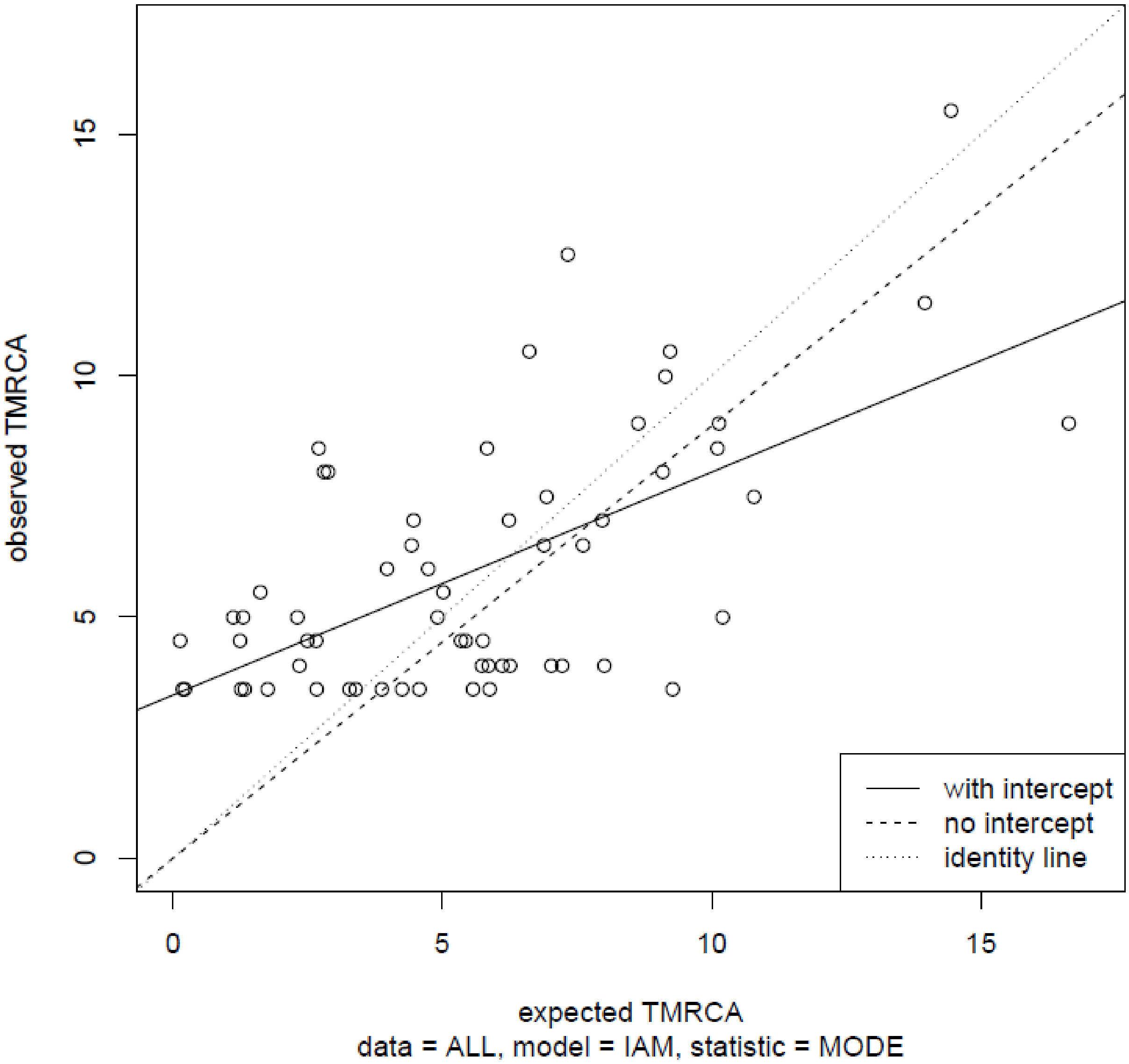

Supplementary Figure S2. Linear regressions (with intercept and without intercept) of observed TMRCA against expected TMRCA for the best fitting case, i.e. using the complete dataset (All), the IAM model and the mode as summary statistic.

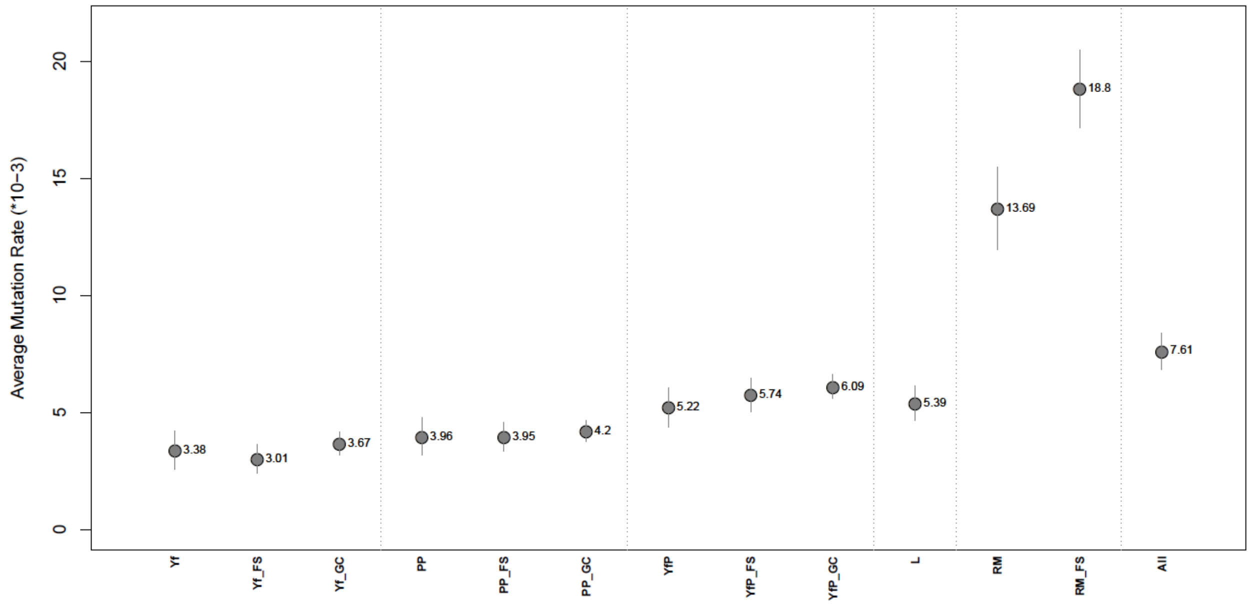

Supplementary Figure S3. Overall mutation rates and 95% confidence intervals for the complete dataset and the considered Y-STR panels (abbreviations as in Table 1) compared with reference values. FS = Father-Son pairs[20] GP = Genealogical Pairs[21].

Supplementary Tables

Supplementary Tables S1-S4 are provided as an Excel file.

Supplementary Table S1. Absolute and relative frequencies of the observed haplogroups in the new sample of 135 individuals belonging to 66 paternal lineages from Emilia Romagna and Veneto.

Supplementary Table S2. Numbers of observed mutations for each considered Y-STR in the new sample of 135 individuals belonging to 66 paternal lineages from Emilia Romagna and Veneto (after excluding potential non-paternity events). Multi-step mutations are detailed, as well as the total counts considering all mutations as single events (Single-step) or multi-step mutations as the sum of independent single-step events (Multi-step). SC = Single-copy Y-STR; MC = Multi-copy Y-STR.

Supplementary Table S3. Diachronic changes of overall mutation rates and 95% confidence intervals for the complete dataset and the considered Y-STR panels (abbreviations as in Table 1) on three increasing bins of meioses (7-10, 11-19, >19).

Supplementary Table S4. Observed TMRCAs (in generations) along with mean, median, mode and 95% confidence intervals of the posterior distributions of estimated TMRCAs using the Walsh[32] procedure. Values are calculated for each of the considered pairs of genealogically related individuals using the complete dataset (All) and the RM and L panels (abbreviations as in Table 1), and fitting the Infinite Alleles Model (IAM) and the Stepwise Mutation Model (SMM). Values in italic correspond to the detected non-paternity events; values in bold correspond to estimates for which the observed value falls outside of the confidence interval.

Supplementary Text

Supplementary Text S1. List of the typed Y-SNPs.

1	L1086
2	L1085
3	V148
4	V168
5	M31
6	P108
7	L419
8	V50
9	M32
10	V42
11	M168
12	P257
13	M174
14	M91
15	P97
16	M42
17	M203
18	M213
19	M45
20	M181
21	M216
22	M9
23	M215
24	M35
25	M78
26	M81
27	M123
28	V6
29	P72
30	M224
31	M136
32	V12
33	V22
34	V65
35	M34
36	P15
37	P16
38	P18
39	M286
40	U8
41	U16
42	U1
43	U13
44	M377
45	M253

46	M21
47	M227
48	P109
49	P259
50	M72
51	P215
52	P37.2
53	M359
54	M26
55	M161
56	M223
57	M284
58	M379
59	P78
60	P95
61	M267
62	M62
63	M365
64	M390
65	P56
66	P58
67	M367
68	M369
69	M172
70	M410
71	M47
72	M67
73	M68
74	M137
75	M158
76	M289
77	M318
78	M319
79	M339
80	M340
81	M419
82	P81
83	P279
84	M92
85	M327
86	M166
87	M12
88	M205
89	M241
90	M99
91	M280
92	M321
93	P84
94	M20
95	M27
96	M317

97	M357
98	M242
99	P36.2
100	MEH2
101	M378
102	SRY10831.2
103	M17
104	M56
105	M157
106	M287
107	P98
108	PK5
109	M343
110	P25
111	M18
112	P297
113	M335
114	M73
115	M269
116	P310
117	U106
118	P312
119	M37
120	M65
121	M153
122	SRY2627
123	M222
124	U152
125	P66
126	M160
127	M70
128	L208
129	M320
130	P77
131	Z18
132	Z381
133	L48
134	U198
135	Z195
136	M529
137	L2
138	L20
139	M60
140	M145
141	M147
142	M96
143	M89
144	M282
145	M69
146	M522
147	M258

148	M304
149	M526
150	P308
151	P79
152	P261
153	P256
154	M231
155	M175
156	P202
157	P326
158	M412
159	L11
160	M124
161	M173
162	M207
163	M281
164	M516
165	M479
166	V13

Supplementary Text 2. R script for performing the Walsh[32] procedure with the Infinite Alleles Model (IAM) and the Stepwise Mutation Model (SMM).

The script, along with a short manual and an example dataset, is provided as a supplementary data file named walsh.zip.
